# Evaluation of the Transition-to-Practice Arrangements for Novice Perioperative Nurses: Protocol for a Mixed Methods Study

**DOI:** 10.2196/64970

**Published:** 2025-01-23

**Authors:** Nick Nijkamp, Pauline Calleja, Ashlyn Sahay, Leanne Jack

**Affiliations:** 1 School of Nursing, Midwifery, and Social Sciences CQUniversity Bundaberg Australia; 2 College of Healthcare Sciences James Cook Univeristy Townsville Australia; 3 School of Nursing, Midwifery & Social Sciences CQUniversity Brisbane Australia

**Keywords:** transition to practice, perioperative nursing, novice nurses, nurse educators, mixed methods research, protocol, document analysis, semistructured interviews, pedagogy

## Abstract

**Background:**

Transitioning into the first year of clinical practice as a nurse or changing specialties in the nursing career presents a critical phase for novice nurses characterized by excitement, apprehension, and the phenomenon of “transition shock.” Within perioperative nursing, this transition phase takes on distinctive challenges. However, there is a lack of empirical evidence on transition programs and arrangements.

**Objective:**

This study aimed to evaluate the current transition-to-practice (TTP) arrangements available to new graduate and novice nurses within Australian perioperative nursing settings.

**Methods:**

This study uses an exploratory mixed-method, multilevel triangulation with a sequential phase design to address 4 research questions. Phases 1 to 3 will use document analysis, surveys, and semistructured interviews to establish the findings of the research questions. Phase 4 will use meta-inference and triangulation to aggregate and analyze the data from all preceding phases. These findings will be the foundation for developing a framework to inform future TTP arrangements. This robust framework will embed empirical evidence, existing literature, and sound learning and teaching pedagogy. Results emerging from this study will be reported using the Good Reporting of Mixed Methods Study guidelines.

**Results:**

This project received approval in June 2023. Following this, Human Research Ethics Committee approval was sought for phases 1 and 2, and recruitment began. As of August 2024, phase 1 has collected 50 responses and phase 2 has collected 69 responses. Data collection for phase 3 is projected to commence in May 2025 once data from phases 1 and 2 have been analyzed. Phase 4 is projected to occur in 2026. Each phase is anticipated to have a results manuscript submitted for publication once data are analyzed and written up.

**Conclusions:**

The findings of this study will provide an in-depth exploration of TTP arrangements within perioperative nursing in Australia and provide a framework to guide the future development of TTP arrangements.

**Trial Registration:**

OSF Registries osf.io/zm432; https://osf.io/54s36

**International Registered Report Identifier (IRRID):**

DERR1-10.2196/64970

## Introduction

Transitioning from undergraduate nursing studies or other nursing specialties into perioperative nursing is a pivotal phase in a nurse’s career journey. This critical period in a novice nurse’s career is often filled with excitement, nervousness, and apprehension. It is not uncommon for novice nurses to experience transition shock during this period, a term that describes a state of emotional and physical shock [1]. Novice nurses often describe transition as feeling like they are “drowning”, “terrified,” or “scared to death” [1]. Within the specialized field of perioperative nursing, transition challenges take on a unique dimension. Perioperative nurses play a vital role in surgical settings, where precision, attention to detail, and seamless care coordination are paramount. Professional health care organizations recognize the unique nature of critical care environments, such as perioperative nursing, that require specialized clinical competence and an understanding of the environment [2,3].

Transition shock in novice nurses arises from the sudden shift from a structured, academic environment to the complex and high-pressure demands of real-world practice, which often differ greatly from their expectations [1]. Without the support systems novice nurses rely on as students, novices can feel isolated, anxious, and insecure in their abilities. The disconnect, paired with high responsibility and fear of making mistakes, contributes to self-doubt, stress, and sometimes burnout in the early months of their job [1].

It is common practice for novice nurses to gain employment opportunities that offer the prospect of completing structured transition-to-practice (TTP) arrangements [4]. TTP arrangements serve as vital conduits connecting the theoretical knowledge acquired during undergraduate nursing education with the knowledge and skill requirements of real-world clinical practice. TTP arrangements play a pivotal role in nurturing the growth of novice perioperative nurses by providing mentorship, tailored training, and a supportive environment where new graduates can gain hands-on experience and develop the competence and confidence necessary for successful nursing practice [5,6].

A lack of empirical evidence regarding TTP arrangements within the perioperative nursing setting was noted during the literature search [7]. Transition arrangements and the impacts of transition shock are well defined within the literature [5,6]; however, there was a paucity of empirical literature that discussed the perioperative TTP arrangements. Most of the literature identified were discussion pieces [7]. Second, the “Educating the Nurse of the Future” report described the significant variability between TTP arrangements and their providers [8]. In this report, it was identified that there is no set standard for the length of transition arrangements, the content taught, how the content is taught, or the other supporting mechanisms available to novice nurses.

This study will address the 2 critical gaps in the literature and transition practices, as identified above. This paper provides a detailed overview of the methods to be used and a discussion of how the study will systematically investigate current practices in transition arrangements and the efficacy of these arrangements. It seeks to explore the perspectives of novice perioperative nurses and perioperative nurse educators regarding transition arrangements.

## Methods

### Study Aim

The proposed research study aims to evaluate the current TTP arrangements available to new graduate and novice nurses (hereafter referred to both groups as novice nurses) within Australian perioperative nursing settings and draw on these findings to develop recommendations and a theoretical framework to guide the design, development, and implementation of future transition arrangements within the perioperative nursing specialty.

### Study Objectives

The following research objectives stem from the research aim and have been used to guide the development of the research questions. The research objectives are (1) to develop an understanding of the transition to practice arrangements in the perioperative environment; (2) to engage key stakeholders to share their opinions, expertise, and experiences on transition arrangements within the perioperative nursing environment in Australia; (3) to undertake critical analysis of the transition arrangements available in Australia to support novice nurses entering perioperative nursing; and (4) and to develop a framework and model that provides evidence-based educational, social, and holistic transition arrangements for novice nurses entering perioperative nursing.

### Research Questions

The following research questions (RQs) are designed to meet this study’s aims and objectives:

- RQ1: What are the current TTP arrangements available to novice perioperative nurses within Australian health care organizations?
- RQ2: What are the content, methods, and educational philosophies used within these TTP arrangements?
- RQ3: How effective are TTP arrangements in supporting novice perioperative nurses and guiding them throughout the transition period?
- RQ4: What support do novice perioperative nurses undertaking TTP arrangements require to become competent and confident clinicians?

### Design

**Figure 1:**
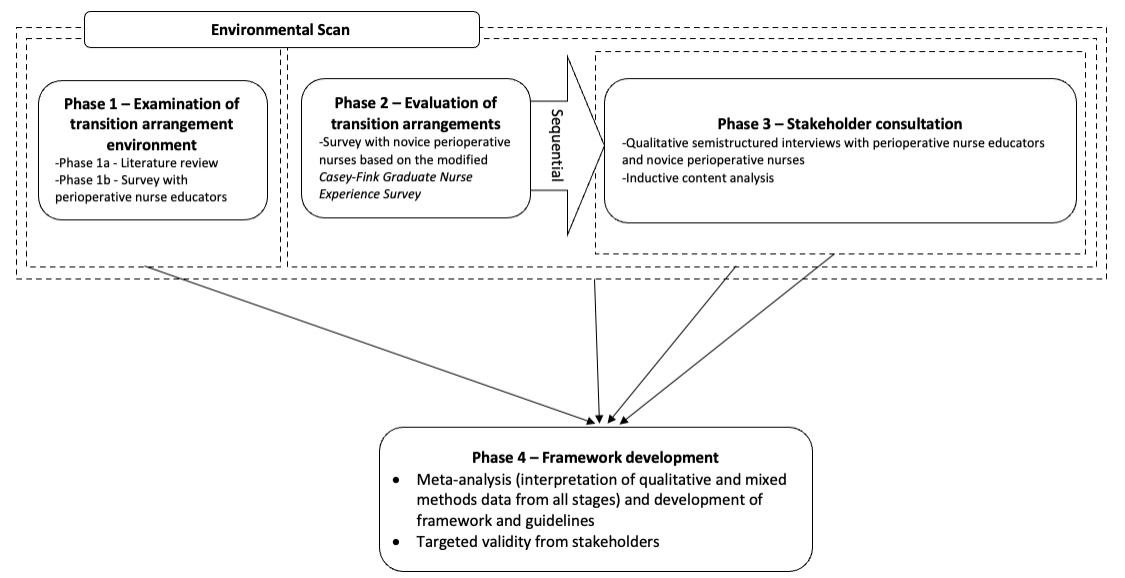
Overview of the exploratory mixed-method, multilevel triangulation design with sequential phases.

This study uses an exploratory sequential mixed-method, multilevel triangulation design (Figure 1). This methodology combines both qualitative and quantitative data to yield a deeper understanding of the research questions [9]. By mixing qualitative (subjective) and quantitative (objective) research, the researcher is provided with the flexibility to explore complex issues and answer multifaceted research questions, thus bridging the divide between the 2 individual research approaches [10]. Several multifaceted research questions need to be answered within this study, which would not be feasible by using only quantitative or qualitative data sources.

This research study has 4 phases (Figure 1). The sequential design will be applied between phase 2 and phase 3 [11]. Phase 1 involves document analysis and surveys to establish the existing TTP arrangements for perioperative nursing in Australia. Phase 2 involves surveys with novice perioperative nurses to determine the effectiveness of TTP arrangements. The findings from these phases will inform the development of interview questions for phase 3. Phase 4 of this study will involve a meta-analysis of all the data collected, which will be used in the development of a framework. An overview of this research design is represented in Figure 1, illustrating the research questions being addressed and the primary method used at each phase. Ethics approval will be sought for phases 1 and 2, as these will run concurrently, and for phase 3.

#### Phase 1: Transition-to-Practice Arrangement Analysis

The first phase of the study will address RQ1 (what are the current TTP arrangements available to novice perioperative nurses within Australian health care organizations?) and RQ2 (what are the content, methods, and educational philosophies used within these TTP arrangements?) using document analysis techniques and a mixed methods survey. As most health care organizations do not publicly publish their transition arrangements, a survey was designed to collect data about TTP arrangements from perioperative nurse educators. The research team had access to their local TTP arrangements; however, surveying perioperative nurse educators will ensure that this study is not geographically confined. Using 2 data collection methods ensures sufficient results are obtained to understand the TTP arrangements used within the Australian perioperative environment.

#### Participants, Sample Size, and Recruitment

Data will be collected from 2 separate sources. First, the document analysis, which seeks to explore and understand TTP arrangements from within Australia. These TTP arrangements will be purposefully selected to ensure completeness and that they are currently used in novice perioperative nurses’ training and transition support.

Second, a survey will be undertaken with a convenience sample of Australian perioperative nurse educators. Sample size calculations indicate an ideal sample size of 28 participants will be required using a confidence level of 95% and a margin of error of 5%) [12] to capture adequate responses to generalize TPP arrangement practices in Australia. Perioperative nurse educators who participate in this survey must be currently used within an Australian perioperative department in a role that allows them to provide nursing education, management, or support to novice perioperative nurses. Participants will be recruited through snowball sampling techniques and social media such as LinkedIn, Facebook (Meta), and Twitter (rebranded as X) [13]. In addition, professional nursing organizations and peak bodies for perioperative nursing will be approached to disseminate the survey.

#### Data Collection and Analysis

Manual searching of gray literature search engines, professional organizations, and health service websites will be used to identify suitable TTP arrangements for the document analysis component of this phase. Data collection for the document analysis and survey will be completed using a fit-for-purpose online survey. The survey tool was designed based on the authors’ scoping literature review findings [7]. The survey instrument will collect data regarding the duration of TTP arrangements, modality, pedagogy and content taught, assessments, efficiency, and barriers. The survey will remain open until the required sample size is reached, or until all recruitment strategies have been used.

The document analysis tool and survey will collect both qualitative and quantitative data. Qualtrics StatsIQ will be used for descriptive statistical analysis and frequencies for quantitative data. Qualitative data will be analyzed using Braun [14] and Clarke’s [15] 6-step guide to thematic analysis. NVivo (Lumivero) software will be used for data management and coding of qualitative data. The findings from this phase will be used to inform the semistructured interviews for phase 3.

#### Phase 2: Survey Phase

Phase 2 of the project will use an online survey to address RQ3 (How effective are TTP arrangements in supporting novice perioperative nurses and guiding them throughout the transition period?) by exploring the effectiveness of TPP arrangements within perioperative nursing from the novice nurse perspective. By doing this, the researcher will understand the efficacy of TTP arrangements within perioperative nursing. Understanding transition practices, particularly from the novice nurse experience, is essential as novice nurses are integral to transition arrangements. Transition arrangements should be designed around novice nurses’ learning and organizational needs and resources.

#### Participants, Sample Size, and Recruitment

A convenience sample of novice perioperative nurses who have recently completed a TTP arrangement within perioperative nursing will be sought for this study phase. It is anticipated these participants will be familiar with the transition arrangement they undertook. A 3-year inclusion criterion is set based on Benner’s Novice to Expert Theory [16]. Nurses within the perioperative specialty with over 3 years of experience are often considered competent and an expert and no longer novices [16].

Significant missing data were noted when attempting to calculate sample sizes for this phase. An accurate estimate of the current population of novice perioperative nurses in Australia was unable to be achieved without making several assumptions that may lead to inaccurate sample size calculations. As the purpose of this phase is to inform the interview development of the next phase (interview phase), therefore a purposive, convenience sample will be the aim for this survey, where all reasonable recruitment strategies will be undertaken and the survey will remain open for 12 months.

Recruitment will be conducted through social media and snowballing, through professional organizations such as the Australian College of Perioperative Nurses, at conferences, and through university contacts to disseminate to graduate perioperative nursing students.

#### Data Collection and Analysis

Data will be collected using the revised Casey-Fink Graduate Nurse Experience Survey [17]. The survey tool was modified with the written permission of the survey tool authors. Adjusting the survey allows contextualization to the Australian perioperative nursing setting and enables the inclusion of further questions relating to participants’ experiences during the transition. The Casey-Fink Graduate Nurse Experience Survey examines novice nurse transition across 8 domains, these being Role Confidence, Organize and Prioritize Care, Support, Role Satisfaction, Stress and Burnout, Resilience, and Organizational Commitment and Preceptorship [17].

Data will be analyzed using SPSS (IBM Statistics) software [18]. Descriptive and inferential statistics will be completed, including reliability testing (Cronbach α), linear regression, and factor analysis (varimax rotation) [19,20]. The NVivo software [15] and Braun and Clarke’s [14] 6 steps of thematic analysis will be used to analyze the open-ended qualitative survey questions. The findings from this phase will be used to inform the semistructured interviews for phase 3.

#### Phase 3: Interview Phase

In phase 3, semistructured interviews will be conducted to address RQ4 (What support do novice perioperative nurses undertaking TTP arrangements require to become competent and confident clinicians?). These interviews will involve 2 cohorts: novice and perioperative nurse educators. The aim of phase 3 is to comprehensively examine the components of transition arrangements essential for success from the perspective of these 2 sample groups. This approach will also facilitate an exploration of the similarities and differences in the perspectives of the 2 cohorts.

#### Participants, Sample Size, and Recruitment

A total of 8-12 participants from each cohort will be sought to complete semistructured interviews. Purposive sampling techniques will be used to ensure a suitable breadth of participants. Continuous comparative analysis will be used throughout the interview process to examine data saturation. Participants must meet the eligibility requirements of novice perioperative nurses and perioperative educators, as defined in the above phases, to participate in phase 3.

Recruitment for phase 3 will commence in the previous 2 phases. Both cohorts will have the option to express their interest in participating in follow-up interviews after the completion of their survey. To maintain the confidentiality of participants and their survey responses, participants who choose to enroll for follow-up interviews will be redirected to a separate survey to collect their names, contact details, and cohort group. The study will use social media and snowball if insufficient participants are recruited through this method.

#### Data Collection and Analysis

A data collection instrument will be developed for phase 3, following the sequential completion of phases 1 and 2. The findings from these earlier phases will inform the development of the interview tool and semistructured interview questions. Before delving into a detailed exploration of TTP arrangements, a comprehensive understanding of the content, methods, and philosophies within TTP arrangements and the experiences of novice perioperative nurses must be obtained.

The interviews will be audio recorded and transcribed verbatim before data analysis is undertaken. NVivo software [15] will be used for thematic analysis using Braun and Clarke’s [14] 6-step guide. This process will be applied to both cohorts, and the results will be compared and triangulated to uncover the experiences and perspectives related to TTP arrangements of novice perioperative nurses and experienced perioperative nurse educators. Differences and similarities between the cohorts will be carefully examined and presented.

#### Phase 4: Meta-Inference and Framework Development

The final phase involves the analysis of data and findings from the previous 3 phases. Data will undergo meta-inference analysis and triangulation to establish the findings directly aligned with the research aims, objectives, and questions. These findings will form the foundation for developing a framework to guide the design, development, and implementation of future perioperative nursing TTP arrangements. This framework will be embedded in the empirical evidence uncovered by this study, existing literature, and adult learning principles. Targeted validity checking will be used to ensure rigor and congruence of the research findings.

### Peer Reviews

This study has completed 2 peer reviews. The initial review occurred during the confirmation of candidature milestone for Doctor of Philosophy students. This review encompassed 2 independent reviews from experts in their field. The second review was conducted as part of the approval process by the CQUniversity Human Research Ethics Committee for both phases 1 and 2 of the study.

### Ethical Considerations

As this study spans 4 phases, ethics approval will be obtained in 2 applications. The initial ethics application encompasses phases 1 and 2 and has been approved by the CQUniversity Human Ethics Research Committee under approval number 0000024139. The subsequent ethics application will be submitted once data analysis from phases 1 and 2 is completed and a semistructured interview protocol has been developed for phase 3.

## Results

This project received approval in June 2023. Following this, Human Research Ethics Committee approval was sought for phases 1 and 2, and recruitment began. As of August 2024, phase 1 has collected 50 responses and phase 2 has collected 69 responses. Data collection for phase 3 is projected to commence in May 2025 once data from phases 1 and 2 have been analyzed. Phase 4 is projected to occur in 2026. Each phase is anticipated to have a results manuscript submitted for publication once data is analyzed and written up.

The results from this study will be disseminated locally and at relevant national and international conferences. The findings from this study will also be disseminated in peer-reviewed nursing journals. Media releases will be undertaken as opportunities arise. In addition, this study will be published and disseminated as a thesis document to meet the requirements of the Doctor of Philosophy program.

## Discussion

### International Significance

In examining the international implications of this study, it is evident that the findings should hold significant relevance on a global scale, reflecting the shared challenges encountered by novice nurses worldwide as they transition into specialized fields [21].

Identifying and exploring gaps in empirical evidence regarding TTP arrangements within perioperative nursing is not unique to Australia; many countries grapple with similar challenges in standardizing and evaluating TTP programs [7]. By addressing these gaps, this study will offer insights that extend beyond national borders, providing a comparative perspective on the effectiveness of transition practices.

Furthermore, the development of a framework and model based on evidence-based educational, social, and holistic transition arrangements has implications beyond Australian perioperative nurses. Components of the framework could be used to inform the development of TTP arrangements internationally. In addition, aspects of this study can be replicated internationally to identify common trends and differences between countries in TTP arrangement practices. The challenges novice perioperative nurses face are likely shared globally, making a well-informed framework adaptable and applicable in different countries to enhance the quality of transition programs.

### Available Evidence

It could be argued that TTP arrangements from other specialty areas could be used within the perioperative nursing realm to support novice nurse assimilation; however, perioperative nursing requires a unique skill set and specialized knowledge that is incomparable to other nursing contexts [3,22]. For this reason, a rigorous literature review was undertaken to establish the existing body of literature related to TTP arrangements within perioperative nursing. The literature review identified a paucity of empirical evidence, with most research on perioperative TTP arrangements consisting of discussion and editorial papers [7]. In addition, most papers described the TTP arrangement they offer rather than provide an evaluation of the TTP arrangements. Papers that evaluated TTP arrangements frequently measured efficacy by participant retention postcompletion [7]. In addition, an Australian national report also identified that TTP arrangements are unregulated and unmonitored [8]. This has led to significant variability between TTP arrangements and their providers.

The lack of empirical evidence and the variability between TTP arrangements provide the rationale and justification of this study. The aims, objectives, and RQs developed are aimed to address these gaps and limitations. It will also provide a guiding framework for TTP arrangement design, development, and implementation within perioperative nursing.

### Methodological Discussion

Integration (with or without method integration) is an essential element within mixed methods methodology [10]. This differs from multimethod research, where various methods are used, but data or data analysis are not required to be integrated [10]. Within this study, mixed methods are adopted as the methodology for a multitude of reasons. Mixed methods are frequently used in evaluation research as the research questions are often multifaceted, such as phases 1 and 2 within this study [9]. The third phase of this project requires data collection from multiple sources, as novice perioperative nurses and perioperative nurse educators are key participants in this topic. The mixed methods methodology is well suited to collecting and mixing multiple data sources from various participant cohorts [9,10].

The mixed methods methodology uses significant formalization approaches to ensure rigor and accuracy of results [10]. Numerous mixed-method designs are available for the researcher to use to provide a pragmatic and appropriate approach to their research. This study uses an exploratory mixed-method, multilevel triangulation design with sequential phase design to achieve the desired research aim and objectives [9,10]. Using this design, the researchers can collect data in distinct phases and sequentially. Each phase of this study requires a differing method due to the lack of perioperative nursing TTP literature. In addition, phase 3 of this study relies on the findings from phases 1 and 2. The findings from phases 1 and 2 are related to the characteristics of TTP arrangements and the experiences and efficacy of these arrangements. Using these findings, an interview tool can be developed for phase 3 to explore TTP arrangements in great depth.

### Implementation Challenges and Limitations

The researchers have identified several potential challenges and limitations that may affect this study. The use of online surveys, generalizability, and the methodology will be discussed herein. By identifying these challenges and limitations in the early phases of the study, mitigation strategies can be enacted to prevent or minimize their impact.

The use of online surveys presents a potential implementation challenge. It is estimated that online surveys attract response rates anywhere from 15% to 60% [23]. Regarding this study, it may suggest that the surveys of phases 1 and 2 may not receive the desired number of participants. The implication of this challenge is minimized as sound plans for participant recruitment have been included in the research methods. In addition, the survey will remain available online until an appropriate number of participants is reached. It is also observed within online surveys that some responses may be fraudulent [23]. Fraudulent refers to multiple responses from the same participant, or the tool might be inappropriately and opportunistically used. This may cause misrepresentation in the data collected and affect the findings. This study overcomes this challenge by ensuring that participants must complete a reCAPTCHA before accessing the survey, placing a cookie on participants’ browsers to prevent surveys from being completed more than once by the same participant, and preventing search engine indexing [12].

Sample size and generalizability considerations have been incorporated into this study and the methods. However, capturing all potential demographic participants and participant experiences in a study is difficult. Furthermore, it is difficult to ascertain the population sizes of the participant cohorts within this study. The researcher used their own experiences to establish an estimated total population size that was used for sample size calculations. Therefore, the components of this study may not apply to all perioperative novice nurses. Generalizability has been addressed by ensuring accurate sample size calculations for the relevant phases of the study. In addition, it has been addressed by the recruitment strategies and analysis strategies.

Finally, the mixed methods methodology presents some unique challenges. In particular, the length of the study can be greater than a single-method study [10]. This is particularly relevant within this study as phase 3 is sequential from phases 1 and 2; therefore, the project length will be increased. In addition, there is potential difficulty in reconciling and triangulating differences in data. Data sets that may emerge from this research could be noncongruent. However, this will lead to further discussion possibilities [10]. A delimitation of this method is that data sets from each phase can often be cross-validated and confirmed, adding to the validity and trustworthiness of the research.

## Abbreviations

RQ: research question
TTP: transition to practice

## Data Availability

Data sharing is not applicable to this article as no data sets were generated or analyzed during the preparation of this protocol.
